# Hydrogels and Their Applications in Targeted Drug Delivery

**DOI:** 10.3390/molecules24030603

**Published:** 2019-02-08

**Authors:** Radhika Narayanaswamy, Vladimir P. Torchilin

**Affiliations:** Center for Pharmaceutical Biotechnology and Nanomedicine, Northeastern University, Boston, MA 02115, USA; narayanaswamy.r@husky.neu.edu

**Keywords:** hydrogels, applications, targeted drug delivery, drug release, hydrophobic drug delivery, clinical translation, versatile platform, administration routes, diverse therapeutic areas

## Abstract

Conventional drug delivery approaches are plagued by issues pertaining to systemic toxicity and repeated dosing. Hydrogels offer convenient drug delivery vehicles to ensure these disadvantages are minimized and the therapeutic benefits from the drug are optimized. With exquisitely tunable physical properties that confer them great controlled drug release features and the merits they offer for labile drug protection from degradation, hydrogels emerge as very efficient drug delivery systems. The versatility and diversity of the hydrogels extend their applications beyond targeted drug delivery also to wound dressings, contact lenses and tissue engineering to name but a few. They are 90% water, and highly porous to accommodate drugs for delivery and facilitate controlled release. Herein we discuss hydrogels and how they could be manipulated for targeted drug delivery applications. Suitable examples from the literature are provided that support the recent advancements of hydrogels in targeted drug delivery in diverse disease areas and how they could be suitably modified in very different ways for achieving significant impact in targeted drug delivery. With their enormous amenability to modification, hydrogels serve as promising delivery vehicles of therapeutic molecules in several disease conditions, including cancer and diabetes.

## 1. Introduction

Hydrophilic polymeric networks that are capable of imbibing huge volumes of water and undergoing swelling and shrinkage suitably to facilitate controlled drug-release are called hydrogels. Their porosity and compatibility with aqueous environments make them highly attractive bio-compatible drug delivery vehicles. Their applications are manifold and for several biomedical needs as they are moldable into varied physical forms such as nanoparticles, microparticles, slabs, films and coatings. USA has been the largest producer of hydrogels and is expected to remain so for a few more years [1]. Hydrogels are promising, trendy, intelligent and ‘smart’ drug delivery vehicles that cater to the specific requirements for targeting drugs to the specific sites and controlling drug release. Enzymatic, hydrolytic or environmental stimuli often suffice to manipulate the hydrogels for the drug release at the desirable site [2]. Like the two sides of a coin, there are also the disadvantages associated with their use. The primary disadvantage in drug delivery would be the hydrophobicity of most drugs. The water-loving polymeric core is probably not very ideal to hold incompatible hydrophobic drugs, which is a challenge since many that are currently used and effective in disease therapy are hydrophobic. The tensile strength of these hydrogels is weak and this sometimes causes early release of the drug before arrival at the target site. The following review discusses on how hydrogels are being manipulated presently for improved targeted drug delivery. The modern trend in which the hydrogels are exploited for drug delivery are covered.

The attractive physical properties of hydrogels, especially their porosity, offer tremendous advantages in drug delivery applications such as sustained release of the loaded drug. A high local concentration of the active pharmaceutical ingredient is retained over a long period of time via a suitable release mechanism controlled by diffusion, swelling, chemical or based on some environmental stimuli. 

**Figure 1:**
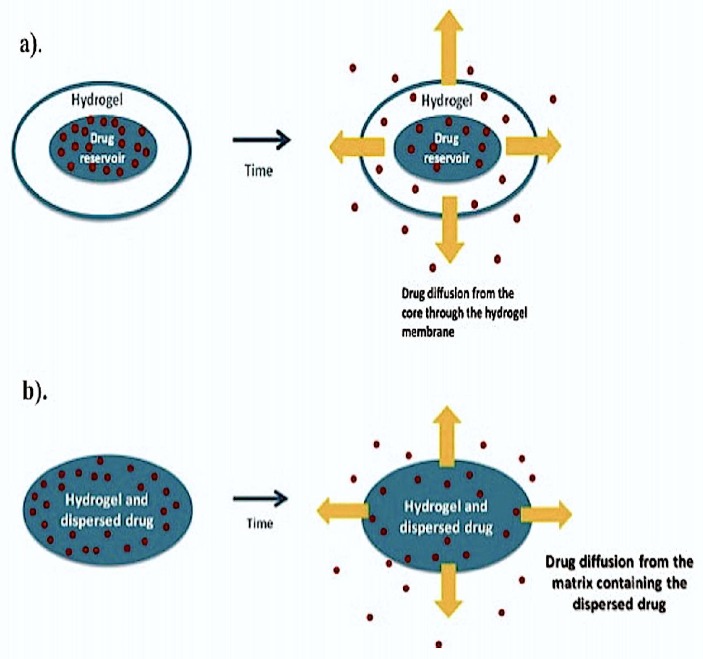
(**a**). Drug-containing core is coated with hydrogel membrane and the drug concentration is higher in the center of the system to allow constant release rate of the same in reservoir delivery system. (**b**). Uniform dissolution or dispersion of the drug throughout the 3D structure of the hydrogel is achieved using matrix delivery (reprinted (adapted) with permission from [3]; the article is open access and the content reusable).

Diffusion-controlled drug delivery with hydrogels uses reservoir or matrix devices that allow diffusion-based drug release through a hydrogel mesh or pores filled with water. In the reservoir delivery system, the hydrogel membrane is coated on a drug-containing core producing capsules, spheres or slabs that have a high drug concentration in the very center of the system to facilitate a constant drug-release rate. While the reservoir delivery system produces time-independent and constant drug release, the matrix system works via the macromolecular pores or mesh. This type of release is time-dependent drug release wherein the initial release rate is proportional to the square root of time, rather than being constant (Figure 1). 

The swelling-controlled drug release from hydrogels uses drugs dispersed within a glassy polymer which when in contact with a bio-fluid begins swelling. The expansion during swelling occurs beyond its boundary facilitating the drug diffusion along with the polymer chain relaxation. The process, otherwise referred to as Case II transport, supports time-independent, constant drug release kinetics. Since the gradient between the dispersed drug in the hydrogel and its surrounding environment allows the active ingredient diffusion from a region of higher concentration within the hydrogel to a lower one, the process is also referred to as anomalous transport as it combines both the processes of diffusion and swelling for enabling drug release.

Ocular drug delivery carriers have been developed using hydrogels that are covalently crosslinked. These soft, biodegradable hydrogels with high swelling capacity remain in-situ in the lacrimal canal offering greater comfort for the patient. Collagen or silicone that may be used decides if the punctal-plug system could be used temporarily or permanently, respectively. Poly(ethylene glycol) hydrogels are commonly used for producing ophthalmic drug delivery systems. 

Drug release in response to environmental changes would be an ideal delivery system as the release becomes very controlled and non-specific side effects at off-target sites are alleviated. Thus, sensitive drug delivery devices responsive to changes in pH, temperature, ionic strength or glucose concentration have been developed that are advantageous in the therapy of diseases such as cancer, and diabetes, characterized by local physiological changes specific to the various disease stages. The polymer composition of the hydrogel responsive to the environmental stimuli is manipulated to make it responsive to the environment [3]. 

Hydrogels considerably enhance the therapeutic outcome of drug delivery and have found enormous clinical use. The temporal and spatial delivery of macromolecular drugs, small molecules, and cells have greatly improved through hydrogel use for drug delivery [2]. Drug delivery using hydrogels, however, has not been free of challenges, but constant improvements are being made to identify the hydrogel design best suited for specific drug delivery purposes. Therefore, this paper discusses the recent trends in drug delivery applications using hydrogels, including their translation to the clinic and their applications to successfully deliver hydrophobic drugs. 

## 2. Current Trend in Hydrogel Based Targeted Drug Delivery

### 2.1. Supramolecular Hydrogels

The supramolecular hydrogel system is composed of intermolecular interactions that are non-covalent and has two or more molecular entities held together. The non-covalent cross-linking is a very attractive aspect of these hydrogels as it helps circumvent the problems of limited drug loading potential and drug incorporation for use only as implantables which would be the only possibility with a covalently cross-linked network. Apart from offering the right physical stability for the hydrogels, these achieve drug loading and gelation simultaneously in an aqueous environment without the need for a covalent cross-linking. Recent progress has been made with supramolecular hydrogels using self-assembled inclusion complexes between cyclodextrins and bio-degradable block copolymers that provide sustained and controlled release of macromolecular drugs [4].

Natural cyclic oligosaccharides composed of six, seven or eight D-(+)-glucose units linked by D-(+)-1,4-linkages (termed α-, β- and γ-CD, respectively), are called cyclodextrins and are well-suited for use in supramolecular systems. They offer hydrophobic internal cavities with a suitable diameter and their ability to generate supramolecular inclusion complexes with various polymers make them ideal drug delivery vehicles. 

A recent study involved development of a glycoconjugate prepared by amidation of homopoly-l-guluronic acid block obtained from *D. antarctica* sodium alginate with mono-6-amino-β-CD. The study was aimed at treating Chagas disease caused by *Trypanosoma cruzi*. Lipophilic non-hydroxylated coumarins were loaded into the hydrophobic core of the β-cyclodextrin to render them with trypanocidal activity. Interaction between the carboxylate groups of unconjugated α-l- glucuronate residues with calcium ions was used to produce supramolecular hydrogels of glycoconjugate of homopoly-l-guluronic block fraction (GG) with 6-NH_2_-β-CD. As the *T. cruzi* parasites have only one mitochondrion, it is an ideal target for drugs to manipulate its energy process and apoptosis. Mitochondrial membrane potential studies revealed that the cyclodextrin complex with the drugs produced significant oxidative stress to destroy the parasites. The drug in the complex had increased solubility, showed improved bio-availability, controlled drug release and improved trypanocidal activity in comparison to the corresponding free amidocoumarins [5]. 

Cyclodextrin-functionalized polyhydrazines were used to prepare hydrogels in-situ via hydrazine bond formation with aldehyde groups on dextran aldehyde. No toxicity was observed *in vitro* with these hydrogels and they could accommodate nicardipine as hydrophobic drug into the cyclodextrin cavities. Steady release of nicardipine over 6 days was observed with the hydrogel preparation having higher hydrazine linkages. Thus, a gel capable of hydrophobic drug release in an in-situ formed device over extended periods was generated [6]. 

**Figure 2:**
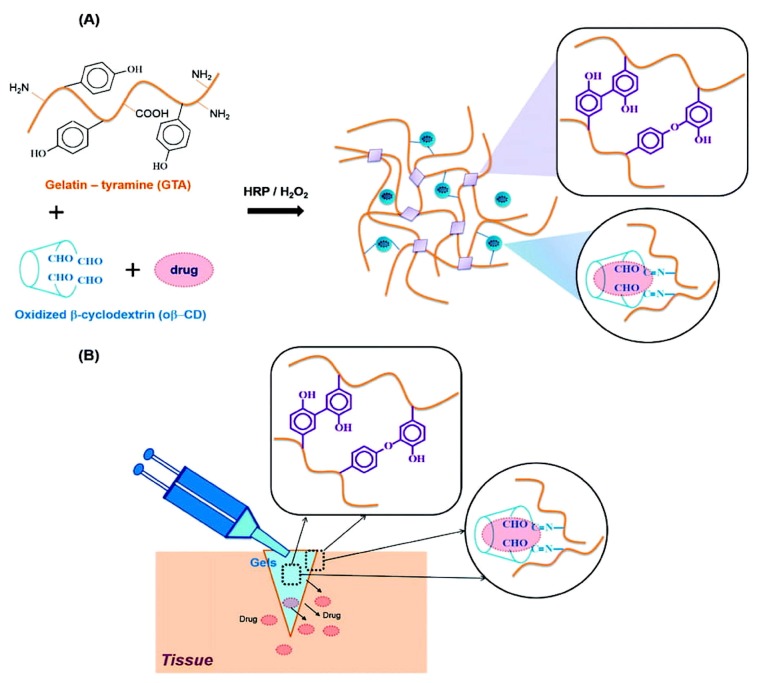
(**A**). Graphical representation of the methods for cross-linking to obtain gelatin-β-cyclo-dextrin (GTA–ob-CD) hydrogels to load hydrophobic drugs. (**B**). Schematic representation of adhesive GTA–ob-CD hydrogels in situ formed by combining HRP catalysis and the Schiff base reaction with therapeutic release (reprinted [7] with permission from the The Royal Society of Chemistry. The article is licensed by Creative Commons and the link to the license is https://creativecommons.org/licenses/by/3.0/).

Bleeding control and wound healing by bio-adhesive hydrogels find enormous biomedical applications. In situ forming hydrogels are used to heal injured tissues based on their ability to accumulate and produce a fibrin bridge that permit fibroblast migration and collagen secretion for healing tissue injury. β-Cyclodextrins are a non-toxic adjuvant for pharmaceutical and mucoadhesive applications. Partly oxidized β-cyclodextrin was used in a recent study to exploit aldehyde groups on a hydrogel matrix for favorable reaction with amines in the tissue to result in an imine bond (Schiff’s base reaction) in order to adhere to the skin and to provide improved cyclodextrin solubility in order to improve loading efficiency. Blending gelatin (the common extracellular component) with the β-cyclodextrin partly oxidized with oxidation in the presence of H_2_O_2_/horseradish peroxidase, resulted in very rapid formation of gelatin-β-cyclodextrin hydrogels (Figure 2). Hydrophobic drugs such as dexamethasone could be released with 2.7 fold higher efficacy when delivered in presence of the cyclodextrin relative to the gelatin-only hydrogels [7].

Curcumin has been shown to have several therapeutic benefits and found enormous applications in conventional therapy. The challenging aspect of its delivery is the extremely low aqueous solubility. However, a glycyrrhetinic acid (GA) molecule-modified curcumin-based hydrogel has been developed to address the problem of delivery of the insoluble drug for hepatocellular carcinoma. The GA molecule-modified curcumin supplied in the pro-gelator form could produce a supramolecular hydrogel *in vitro* due to disulphide reduction by glutathione (GSH) and increase curcumin bioavailability and solubility as reported in HepG2 cells. Higher cellular uptake and potent anti-cancer activity were observed with the hydrogel *in vitro* relative to an already known curcumin-targeting compound that was tested [8]. 

### 2.2. DNA-Hydrogels

Hybrid bionanomaterials could be developed using DNA as the building block. Predictable two- or three-dimensional structures are formed from DNA molecules. Highly structured networks are formed by hybridizing complementary DNA molecules and the resultant hydrogel structures expand upon encounter with an aqueous environment that result in swelling. Not only do these materials append to any other type of nucleic acid molecules (such as siRNA, miRNA), but they can also load DNA binding drugs. High solubility, bio-compatibility, versatility and responsiveness are key features of such hydrogels. Apart from these features they can also be tagged with suitable fluorescent molecules for tracking biological studies *in vitro* [9]. 

**Figure 3:**
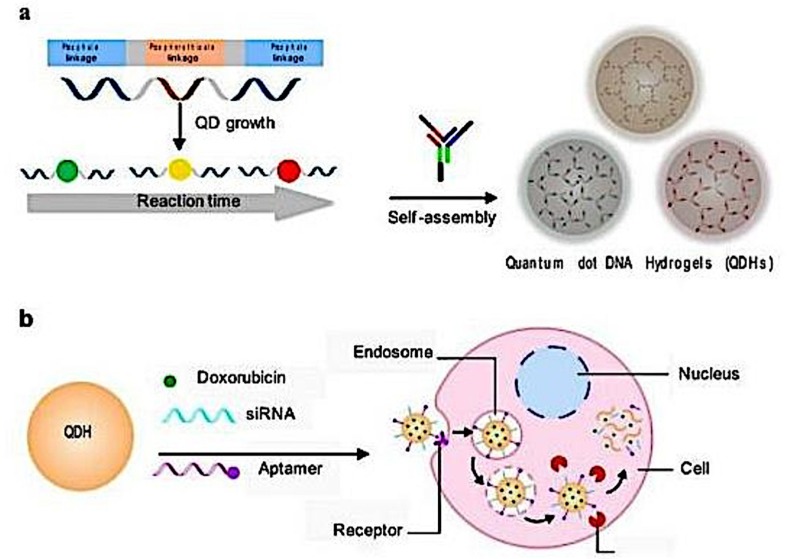
(**a**). Schematic view of the synthesis process of the DNA functionalized QDs followed by the formation of the QD hydrogel through hybridization with DNA. (**b**). Schematic view of the modification process on the QD hydrogel for specific targeting of the cell using aptamer for drug delivery. Release of Doxorubicin and siRNA happens after uptake into the cell via endocytosis (reprinted (adapted) with permission from [9]; the article is open access and the content reusable. Creative Commons International License 4.0 for the article is available from http://creativecommons.org/licenses/by/4.0/) [9].

An interesting application of hydrogels has been made with the development of multi-functional quantum dot (QD) DNA hydrogels. DNA hydrogels are composed of complementary strands of DNA hybridized to form a crosslinked network that swells in an aqueous environment. However, in order for biological studies to be more easily and effectively performed there has to be a tracer or a fluorescent molecule attached to the hydrogel which would then be a superlative option offering both targeted delivery and imaging. With this aspect in mind Zhang et al. recently developed a QD-based DNA hydrogel that had highly tunable size, spectral and delivery properties and bound to the DNA binding drug doxorubicin (Figure 3). The drug targeted cancer cells and the QD DNA hydrogel increased the potency of the drug *in vitro*. The single-step assembling zinc sulphide QD Doxorubicin DNA hydrogels showed increased tumor accumulation *in vitro*, high bio-compatibility, was threefold more efficacious than free DOX and served as an excellent tool for *in vivo* bio-imaging in monitoring tumor growth over time. Aptamers such as siRNA were used to target specific cell types to deliver drug specifically and modulate protein expression in various cell types [9] (Figure 3).

Drug-loaded cytosine-phosphate-guanine (CpG)-DNA hydrogels have been used for cancer immunotherapy. These CpG sequences containing hydrogels elicit immune responses. As an example, these DNA hydrogels containing CpG nucleotides stimulated innate immunity through Toll-like receptor 9 and promoted immune responses of ovalbumin (OVA) incorporated into the gel by acting as an adjuvant. Adverse reactions were considerably reduced with the use of the CpG-DNA hydrogel in comparison to the OVA injected with alum or complete Freund’s adjuvant [10,11].

**Figure 4:**
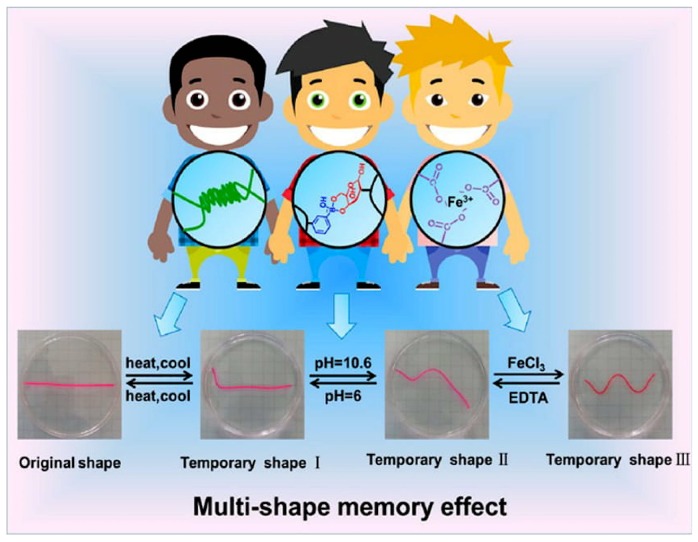
Schematic depiction of a novel Fe^3+^-, pH-, thermoresponsive hydrogel with tunable mechanical properties easily changed by adjusting cross-linking densities of polymers. Moreover, several co-ordination interactions along with supporting stimuli could be exploited to stabilize temporary shapes in order to realize shape memory behavior. Programmable multi-shape memory effect can be realized by combining the 3 reversible switches (reprinted (adapted) with permission from [12]; copyright (2017) American Chemical Society).

There has been significant advancement in hydrogel modifications for enhancing the shape memory and the reversibility of the hydrogel shape after any prolonged stress on them. (example: external stimuli such as temperature). This advances the thermal responsivity behavior of the hydrogels and an example is the construction of supramolecular hydrogel with tunable mechanical properties and multi-shape memory effects. These hydrogels make use of agar that is physically cross-linked to form the hydrogel network and a supramolecular network that is cross-linked by suitable chemical bonds that fix shapes temporarily and produces a multi-shape memory effect made possible by reversible interactions (Figure 4). The supramolecular hydrogel possesses enormous bio-compatibility and bio-degradability characteristics and rely on non-covalent interactions to drive the self-assembly of small molecules in water. The formed structures have supramolecular architectures and encapsulate water [12,13].

A programmed temporary shape can turn into the memorized original shape when placed in an appropriate environment or exposed to a trigger. Such shape-memory hybrid hydrogels could also be synthesized using DNA cross-linkers. These hydrogels not only undergo phase transitions in the trigger of the stimulus, but also possess memory code to recover to the original matrix shape. Guo et al. developed a pH-controlled shape memory DNA hydrogel that was formed by co-polymerization of acrylamide residues with acrydite modified with (1) cytosine-rich sequences (forming i-motif subunits) and (2) nucleic acids exhibiting self-complementarity. Self- assembly of cytosine-rich nucleic acid strands into an i-motif structure occurred at pH 5.0 and the disassembly to a random coil form happened at pH 8.0, leading to a quasi-liquid state for the hydrogel. Re-acidification to pH 5.0 restored the original structure of the gel [14,15]. 

### 2.3. Bio-Inspired Hydrogels

A newer variety of hydrogels used for drug delivery applications are the bio-inspired hydrogels. These 3D materials recapitulate the biological micro-environment relevant to the disease condition and support studies on how the targeted drug delivery process could be optimized, how the therapy behaved *in vivo*, how the disease progressed, and so on. These are particularly useful in cancer therapy as the disease is particularly complex and normally associated with intricate cellular and physiological changes that require progressive monitoring. Engineering such microenvironments would thus be a very useful approach to promote research and study the disease condition and therapeutic process better. The stiffness of the 3D model used for studying liver cancer is a critical attribute to regulate molecular diffusivity and malignancy. The elastic moduli of the collagen gels were increased by stiffening interconnected collagen fibers with varied amounts of poly(ethylene glycol) di(succinic acid *N*-hydroxysuccinimidyl ester). The softer gels produced malignant cancer spheroids while the stiffer ones showed suppressed malignancy. The model provided better understanding and regulation of the emergent behaviors of cancer cells [16]. 

Contact lenses that are bio-inspired have been developed recently with improved drug delivery properties suited to perfectly match the eye condition in the diseased state, especially of the anterior-eye segment. 

Inner layer of ethyl cellulose and Eudragit S 100 hydrogels encapsulated with diclofenac sodium salt showed sustained drug release in simulated tear film. Sustained drug levels in tear fluid for a prolonged period of time in relation to eye drops was observed. The drug timolol was released in response to lysozyme in the medium and not in PBS. Enzyme cleavable polymers in hydrogels enabled this.

Ergosterol-liposome grafted silicone materials loaded with nystatin (an anti-fungal agent) resembled a fungal infection and triggered nystatin release through a competitive mechanism. In the absence of the ergosterol (important sterol in the cell membranes of fungi) in the medium, the drug release was very negligible [17,18,19,20].

With recent progress in cancer therapy using immunology, the research has progressed in the area with the use of cells such as erythrocytes, macrophages, stem cells, dendritic cells and bacteria as building blocks to create targeted delivery systems. Stem cell membrane coated gelatin nanogels that were high tumor targeting and bio-compatible drug delivery systems were developed recently.

Those gelatin nanogels had excellent stability and tumor-targeting ability *in vitro* and *in vivo*. Targeting doxocrubicin (DOX) enhanced anti-tumor therapeutic efficacy significantly higher than the gelatin-DOX of free DOX nanogels. Evident side effects were absent in the tissues of heart, liver, spleen, lung and kidney as was revealed with histopathology staining of treated mice [21]. 

A similar bio-mimetic drug delivery carrier is the endosomal membrane-coated nanogel extracted from the source cancer cells for specific delivery of the small molecule drug. Hyaluronic acid nanogel composed of SiO_2_/Fe_3_O_4_ nanoparticles formed the inner core of the hydrogel and the endosome membrane outer shell was used. The hyaluronic acid could target CD44 receptors overexpressed in a variety of tumor types. Core-shell mesoporous silica nanoparticles with Fe_3_O_4_ nanocrystals were used as the core to encapsulate photo-initiators and cross-linkers, followed by hyaluronic acid coating on the surface via electrostatic interaction. Photo-polymerization upon UV irradiation resulted in the in situ formed nanogel in endosomes following incubation of resultant nanoparticles with the source cells. In the presence of the loaded anti-cancer drug doxorubicin, the endosomal membrane nanogels could specifically interact with the target source cells followed by internalization to release doxorubicin. High targeting specificity, cytotoxicity and uptake of the prepared endosomal membrane coated nanogels with DOX over the bare DOX-nanogels was observed *in vitro* [22]. 

Detoxification of pore-forming toxins resulting from animal bites/stings using a 3D bio-inspired hydrogel matrix resembling the 3D structure of liver is a recent achievement. The 3D matrix resembling the liver is created by the hydrogel while the polydiacetylene nanoparticles installed in the hydrogel matrix serve to attract, capture and sense toxins. The 3D printed biomimetic detoxification device with installed nanoparticles would be a breakthrough innovation replacing vaccines, monoclonal antibodies, antisera to eliminate toxins from blood. Commonly used conventional antidote molecules target specific epitope structures on the pore forming toxins warranting specific treatments for the various toxins. IV-nanoparticle administration for binding and removal of toxins may cause secondary risk by poisoning from nanoparticle accumulation in liver. While challenges do not limit nanoparticle use for toxin clearance, the 3D printed *in vitro* devices have been clinically approved for toxin removal [23].

Adhesive bio-inspired gels comprising of sodium alginate, gum arabic, and calcium ions have been developed in order to mimic properties of natural sundew-derived adhesive hydrogels. Mouse adipose-derived stem cells were used in combination with the sundew-inspired hydrogel to confer interesting properties such as improved wound healing, enhanced wound closure, less noticeable toxicity and inflammation. Sundew plant’s natural adhesive hydrogels were quite promising, but the researchers were quite intrigued about collecting the desirable quantity from the natural source. This inspired development of the above described hydrogel type with improved properties [24]. 

Magnetically actuated gel-bot (Mag-bot) is yet another recent discovery that facilitates remote control of the motion of magnetically actuated hydrogels. These hydrogels adopt a crawling motion resembling that of a maggot and move in a confined space of 3D porous media including, foams, tissues, fibres and so on, opening up broad options for targeted drug delivery. Pattern structures and lubrication effects on hydrogel mobility in the confined 3D space have been experimented in the paper [25]. 

It is not just the maggot that has inspired development of a new generation of hydrogels with applications in targeted drug delivery but also the drosera. The drosera-inspired model of hydrogel for targeted drug delivery with a bifunctional attribute to it has been developed recently. These hydrogels use the ‘catch and kill prey’ mechanism as a drosera would. They have a bottom layer functionalized with double stranded DNA that can bind the anti-cancer drug doxorubicin and there are aptamers on top that offer targeting advantages to these particles. Though there is requirement for *in vivo* studies, the sustained expression of specific aptamer on surface and the continuous killing of cancer cells by sustained drug release make these hydrogels an ideal targeted drug delivery vehicle for killing cancer cells [26]. 

### 2.4. Multi-Functional and Stimuli-Responsive Hydrogels

Hydrogels that were multi-functional and carriers of anti-cancer drugs are a typical example of the versatility of these delivery vehicles and their amenability to chemical modifications to enhance their therapeutic effects. Magnetite nanoparticles that supported increased intracellular uptake by HeLa cells also had folate ligand on them to enable targeted delivery. Moreover, the hydrogel polymers were thermally responsive and DOX loaded. Those modified hydrogels offered advantages such as increased cellular uptake and apoptotic activity *in vitro* [27]. 

**Figure 5:**
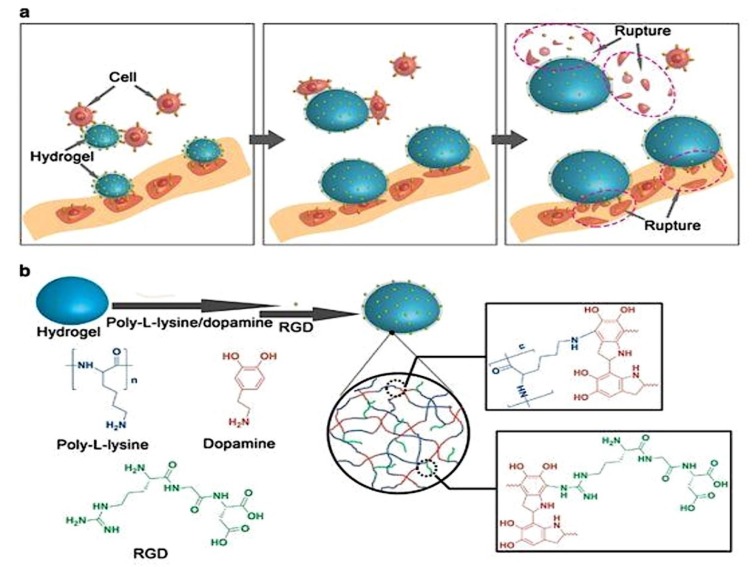
(**a**). Schematic illustration of the cancer cell attachment on stimuli-responsive hydrogel surface. External stimulus such as temperature causes expansion of the hydrogels and rupture of the cancer cells. (**b**). Schematic depiction of the surface modification of hydrogel. The first layer of coating was polydopamine (PDA) and poly-l-lysine (PLL) followed by a layer of RGD peptides for binding cancer cells (reprinted (adapted) from [28]; the article is open access and the content reusable. Creative Commons International License 4.0 for the article is available in https://creativecommons.org/licenses/by/4.0/) [28].

Another stimuli-responsive hydrogel developed very recently made use of biocompatible thermally responsive polymers that facilitated rupture of cancer cells. The study was conducted *in vitro* with an external source of heat and showed successful cell rupture. Those hydrogel particles had RGD (Arginine, Glycine, Aspartate) peptides attached to their surface that could bind to cells (Figure 5). The paper discussed experiments performed to confirm firmness of the RGD peptides with the cells (ex: MDAMB21) and their rupture using external heat stimulation. 

Since *in vivo* conditions in tumors normally produce a higher temperature than the surrounding areas, and also that it has been proved that the physical force generated from the expansion of the cells when they were heated was sufficient to kill them, that type of thermally responsive hydrogels proved to be highly advantageous options for achieving targeted cell killing [28].

**Figure 6:**
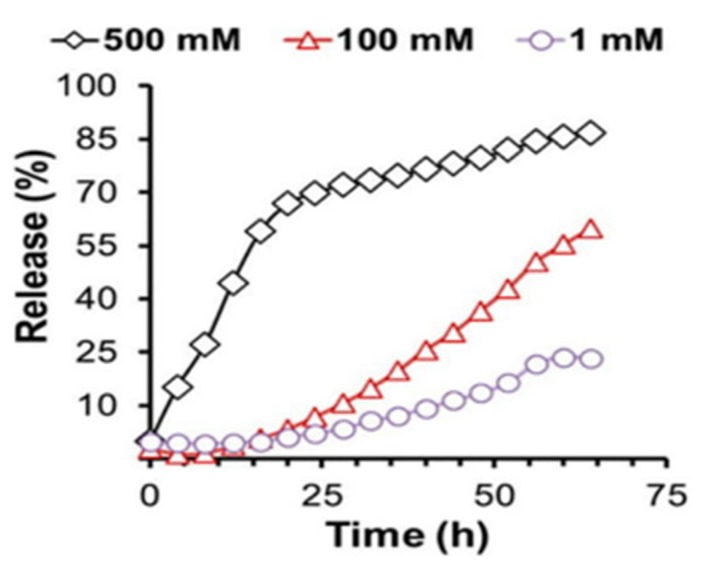
Drug release dependent on Reactive Oxygen Species concentration as displayed by PPS_60-_b-PDMA 150-b-PNIPAAM150 triblock polymer-based thermos-responsive Nile red-loaded hydrogels. Nile red-loaded hydrogels (5 wt% triblock copolymer concentration) in PBS (pH 7.4) have been used to demonstrate Hydrogen Peroxide dependent drug release kinetics *in vitro* at 37 °C. 1, 100 and 500 mM concentrations of H_2_O_2_ over a 64 h time course were incubated with the hydrogel samples to study ROS dependent drug release (reprinted (adapted) with permission from [31]; copyright (2014) American Chemical Society).

Hydrogen peroxide trigger-mediated release of drugs could be achieved using hydrogels. This has been demonstrated previously with the use of ABC type triblock copolymer poly [(propylenesulfide)-*b*-(*N*,*N*-dimethylacrylamide)-*b*-(*N*-isopropylacrylamide)] [29,30] (PPS-*b*-PDMA-*b*-PNIPAM). A model hydrophobic drug, the dye red Nile was encapsulated in the hydrogel in order to demonstrate this. The gelation to form the cross-linked hydrogel happened above 37 °C and the ambient 25 °C temperature was sufficient to encapsulate the hydrophobic drug through the formation of micelles. Thus, the temperature-responsive polymer could cross-link to be a stable hydrogel carrying the hydrophobic drug based on the temperature modification and also showed a release of the red colored dye based on the H_2_O_2_ release in the environment (Figure 6).

Apart from the polymer solubility switch mechanism that helped release based on the presence of the reactive oxygen species in the environment, cleavable units of polymer that underwent scission in presence of ROS have also been developed. These are also bio-degradable and help avoid any toxic build up or unwanted immune response in the system from the presence of the hydrogels [31,32]. 

Diselenide-containing block copolymer and a peptide amphiphile were used as building blocks for the construction of a UV responsive hydrogel. The gel sol transition of the hydrogel occurred in presence of gamma irradiation. The peptide amphiphile was composed of nalproxen, the drug and a hexapeptide sensitive to UV irradiation, the radiation trigger was sufficient to cause release of the drug from hydrogel. The stimuli responsive hydrogel system was thus capable of providing both chemo- and radiotherapy benefits on application [13]. 

Covalently cross-linked hydrogels are typically stable and elastic while the physically cross-linked hydrogels produced through ionic interactions are less stable and exhibit reduced mechanical properties. The dynamic covalent chemistry is an option to acquire stability, elasticity and also shear-thinning and self-healing characteristics for the hydrogel. Based on this, Volkan et al. reported the synthesis of hydrogel networks from reversible interactions between phenylboronic acid and *cis*-diols. The gel strength was dependent on pH and the hydrogel was evaluated for protein delivery *in vitro*. The pKa of phenylboronic acid and the pH of the environment were the determining factors for establishing the extent of hydrogel cross-linking. Soft, moldable hydrogels were produced that could be injected using standard syringe needles and were demonstrated to exhibit shear thinning and healing properties in addition to their injectable nature. Protein encapsulation was performed with the hydrogel, and the release effect monitored. Size-dependent protein release could be attributed to the mesh size of the hydrogel network. Insulin and IgG release from the hydrogel network was monitored in presence of glucose. Glucose-responsiveness of the hydrogel was confirmed, and the release kinetics could be controlled by the mesh size of the hydrogel. 3T3 fibroblast cells showed no significant toxicity as quantified using MTT assay for 24 hr. No chronic inflammation was observed *in vivo* and the materials were quite bio-compatible [33].

The temperature sensitive hydrogels respond by undergoing sol-gel transitions with changes in temperature from room ones to the physiological ones. Recently developed hydrogels were that were thermo-responsive and accommodated hydrophobic drugs efficiently were built from amphiphilic triblock co-polymers, poly(*N*-isopropylacrylamide)-*b*-poly(4-acryloylmorpholine)-*b*-poly(2-((((2-nitrobenzyl)oxy)carbonyl)amino)ethylmethacrylate)(PNIPAM-*b*-PNAM-*b*-PNBOC). The hydrogel carried the hydrophilic drug gemcitabine and the hydrophobic drug doxorubicin. The triblock co-polymers first assembled into micelles that had the hydrophobic and temperature responsive components formed below the lower critical solution temperature, while higher polymer concentration and temperature above the critical gelation temperature were used to form the hydrogels of physically cross-linked micellar nanoparticles. The study demonstrated the synthesis, characterization and the temperature and UV irradiation triggered synergistic release of both the hydrophobic and hydrophilic drugs *in vitro* [34,35].

Hydrogels formed by covalent cross-linking of polymers to facilitate targeted drug delivery have been achieved in recent years. The nanoparticle-hydrogel is a hybrid system that is formed in three different ways. The first method is to entrap a hydrogel within a nanoparticle, the second one is to form a 3D hydrogel network with the nanoparticles by crosslinking of the latter using hydrophobic interactions or mixing up nanoparticles of opposite charges and the final one is to covalently couple the nanoparticle with the hydrogel [36]. A liposome cross-linked hybrid hydrogel has been developed recently. Glutathione-triggered release from the stimuli responsive polymer favored drug release. Arylthioether succinimde cross-links were introduced between the Peg polymers and the liposome nanoparticles to produce the 3D hydrogel network. In presence of glutathione, the matrix was degraded, and the encapsulated drug molecules were released. Malemimide-functionalized liposomes crosslinked using peg polymers were constructed for co-delivery of doxorubicin and cytochrome-C (apoptotic cascade initiatior) and their release testing *in vitro* was monitored in presence of glutathione. The presence of glutathione is a trigger for release from the hydrogel in a reducing microenvironment such as a tumor [37]. 

## 3. Diverse Physical Attributes of Hydrogels for Drug Delivery

Structural-modification amenability of the hydrogels renders them in various shapes and sizes. This feature is particularly interesting for drug delivery applications in order to design the hydrogels as per the target sites to which the drugs have to be delivered. The hydrogel-based dosage forms can have different designs and shapes depending on the route of drug administration (Table 1).

## 4. Specific Therapeutic Areas Using Hydrogels for Drug Delivery at Present

### 4.1. Ophthalmic 

Conventional eye drops have problems with sustained drug delivery and there is huge wastage of drug immediately following application through eye drainage. Dextenza is a very recently FDA approved (3 December 2018) ocular therapeutic hydrogel formulation for human use. This is used for ocular pain following ophthalmic surgery and is the first intracanalicular implant developed for drug delivery and developed by the company Ocular Therapeutix (Bedford, MA, USA) [51].

Thermo-responsive polymer developed by mixing poly(acrylic acid-graft-*N*-isopropylacrylamide) (PAAc-graft-PNIPAAm) with PAAc-co-PNIPAAm geL and incorporating epinephrine was used in the *in vitro* evaluation of ophthalmic drug release. The approach augmented the effect of intraocular pressure reduction from 8 h with the traditional drops to 36 h. The cross-linking density of the hydrogel affected the capillary network formation and offered a convenient controlled drug release method for ophthalmic drug delivery [52]. 

Intra-ocular pressure (IOP) elevates during glaucoma and alleviating this pressure has been quite challenging. Hydrogels could be used to resolve this problem by using them to prepare soft contact lenses composed of polymers to form networks. The highly hydrated polymer networks of hydrogels cause the drug to elute out very rapidly and this is not favorable for glaucoma therapy, which mainly uses hydrophilic drugs. However, with suitable modifications, soft contact lenses have been developed using polymers of *N*,*N*-diethylacrylamide and methacrylic acid, which delivered the hydrophilic drug timolol for about 24 h, thereby opening up ways to allow sustained hydrophilic drug delivery using hydrogels. Storing the contact lenses in a hydrated state can leach out drug and to wear them all the time are the limitations though [53]. 

Inner layer-embedded contact lenses have been investigated for the sustained release of highly water-soluble drug betaxolol hydrochloride on the ocular surface. Cellulose acetate and Eudragit S-100 were selected as the inner layer of the contact lenses which showed a promising sustained drug release for over 240 h in tear fluid of rabbits *in vivo* to create a controlled-release carrier of the drug in ophthalmic drug delivery [19]. 

Controlled drug release behavior from hydrogels was also evaluated using nepafenac as the model drug. 3D cross-linked thermos and pH sensitive hydrogel was designed that was composed of carboxymethylchitosan (CMC) and poloxamer with glutaraldehyde as the cross-linking agent. The hydrogel was found to undergo reversible sol-gel transition at temperature and/or pH alteration at a very low concentration. Sustained release of the drug nepafenac was observed in the *in vitro* model and maximum release was observed at 35 °C and pH 7.4. Cytocompatibility of the hydrogel with human corneal epithelial cells was high [54].

### 4.2. Oral, Intestinal 

Gastroretentive drug dosage forms (GRDDFs) are particularly attractive for drugs that are absorbed in the proximal part of gastroinstestinal tract. Enhancing the retention time of the drugs in the GI tract is very important in order to improve their bioavailability and enhance their therapeutic effects. These dosage forms could be exploited for their muco-adhesion to the gastric mucosa, modified to float or sink in order to prevent leaving the stomach or increase their swelling behavior and make them as large to prevent passage through pylorus for prolonged periods. Based on these ideas, polyionic complex hydrogels of chitosan with ring-opened PVP have been developed for Osteoporosis therapy. The formulation was used to release alendronate in the upper GI tract. Enhanced muco-adhesion, delayed clearance from swelling, minimal localized irritation, improved bioavailability and slower release of the active ingredients are the interesting aspects of the preparation. Also, *in vivo* experimentation showed that these hydrogels could provide optimized PK properties that maintained the drug in the therapeutic levels for a sustained period of time, minimizing fluctuations in therapeutic levels, hence also the possible side effects [55]. 

Inflammatory diseases such as irritable bowel syndrome have been recently treated using hydrogels. These provided safer alternatives to delivery methods that may cause systemic toxicity. Zhang et al. developed negatively charged hydrogels that preferentially accumulated in the positively charged inflamed colon and acted as carriers of the corticosteroid drug dexamethasone (Dex). The hydrogel was prepared from ascorbyl palmitate which had labile bonds responsive to inflammatory conditions and was Generally Regarded as Safe (GRAS) for administration. Enema administration to the colon of inflammation targeting (IT) hydrogel microfibers not only reached the target site but also stayed there owing to charge interaction. The formulation was therapeutically very efficacious and revealed lesser systemic drug exposure than with free Dex in the IBS mice model *in vivo* [56]. 

Complexation hydrogel prepared from poly (methacrylic acid-g-ethylene glycol) [P(MAA-g-EG) has been described. The targeting ligand used is the octarginine cell-penetrating peptide that causes specific delivery of insulin to the intestine. This method facilitated ideal targeting, absorption at target and allowed immediate release of insulin from absorption site. Great hypoglycemic responses were achievable and increased insulin absorption was noted from diabetic rat models used for testing. 18% glucose reduction was observed immediately on administration of the hydrogel containing insulin [57]. 

### 4.3. Cardiac Illness and Cancer

Myocardial infarction is a leading cause of death and disability in the world. Intramyocardial administration of biomaterials such as hydrogels along the perimeter region of myocardial infarction has proven to be beneficial. 

Chen et al. proposed the use of a combination of curcumin (known for its anti-oxidant, anti-inflammatory and anti-oxidation properties) and nitric oxide (known as an anti-angiogenesis agent) in a hydrogel to treat myocardial infarction. The mixed component hydrogel created with the combination drugs improved therapeutic efficacy synergistically. Protective effects such as myocytic apoptotic death alleviation, reduced collagen deposition, increased vessel density (attributable to NO in the combination) and upregulated Silent Information Regulator 1 (SIRT-1), a histone deacetylase that confers resistance to the heart from ischemic injury were observed in diseased mice models *in vivo*. The hydrogel was prepared using peptide derivatives of curcumin and NO in a ratio of 4:1 and showed sustained curcumin release at a low concentration of 2.5 μg per ml per 24 h. NO was released in presence of the enzyme β-galactosidase that could break glucosidic bonds to release NO [58]. 

Growth factors and cytokines (paracrine factors) secreted by stem cells have been proven to be effective in repairing damaged myocardial tissue. The whole cocktail of the paracrine factors is referred to as a secretome and is isolated *in vitro*. The biomolecular composition of the secretome can be manipulated suitably by varying stem cell culture conditions. An injectable hydrogel to deliver to peri-infarct myocardium has been recently developed using secretome from human adipose derived stem cell secretome. Nano-composite hydrogel was formed from a combination of gelatin and laponite carrying the secretome and tested both *in vitro* and *in vivo* for their therapeutic effects via monitoring angiogenesis, scar formation and heart function. Significantly reduced scar area and improved cardiac function were observed *in vivo* in the secretome loaded hydrogel group in relation to the control [59]. 

The very recent development of a paintable hydrogel to serve as cardiac patch for treating myocardial infarction is worth mentioning in this context. The hydrogel eliminates the damage to tissue through suture or light triggered reactions as it is paintable. It has been constructed by a Fe^3+^ triggered polymerization reaction wherein the covalently linked pyrrole and dopamine undergo simultaneous polymerization with the trigger and the conductive polypyrrole produced also uniquely cross-links the network further. The functional patch is both adhesive and conductive and forms a suture-free alternative for reconstruction of cardiac function and revascularization. Bonding within 4 weeks to the beating heart boosts the transmission of electrophysiological signals with conductivity profiles equivalent to that of the normal myocardium [60]. 

Bio-material based immunotherapy platforms for targeted drug delivery to cancers are the latest trend observable in cancer therapy. Based on this idea, novel STINGels have been developed by Leach et al, that are peptide hydrogels to show controlled delivery of cyclic dinucleotides (CDns). Dramatic improvement in survival was observed in murine models of head and neck cancer in comparison to CDN alone or CDN delivered from a collagen hydrogel [61]. 

**Figure 7:**
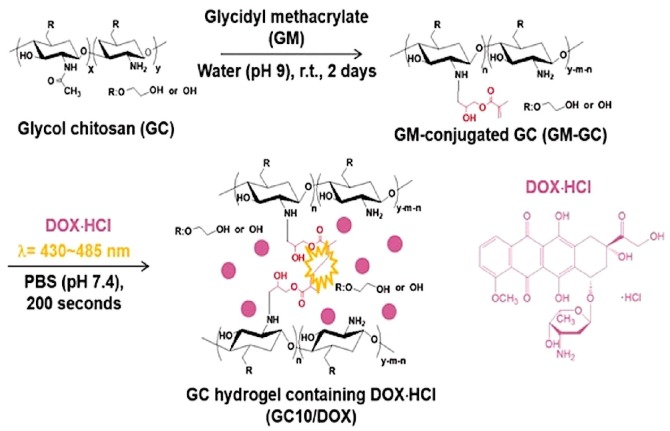
To glycol chitosan solution glycidyl methacrylate (GM) was added in water (adjusted to pH 9) and maintained for 2 days at room temperature. The white solid conjugate of GM was dissolved in water and riboflavin added. DOX·HCl was added and the mixture was irradiated using blue visible light (430–485 nm, 2100 mW/cm^2^) for 10 minutes in order to promote hydrogelation (reprinted with permission from [62]; the article is open access and the content reusable. Creative Commons International License 4.0 for the article is available in https://creativecommons.org/licenses/by/4.0/).

Thyroid cancer treatment using local drug delivery system formed of glycol chitosan (GC) hydrogel and doxorubicin hydrochloride (DOX·HCl) called GC10/dox has been recently developed (Figure 7). Visible light regulated the storage and swelling aspects of the hydrogel and a controlled sustained release followed the initial burst release within 18hours. Potent antitumor effects were observed *in vivo* and *in vitro* in comparison to free DOX·HCl and this is a promising research direction for thyroid cancer therapy [62]. 

Injectable hydrogels responsive to Reactive Oxygen Species that degrade in the presence of ROS and promote immunogenic tumor phenotype via local gemcitabine delivery is a recent discovery. The PVA cross-linked hydrogel with ROS-labile linkers enhance anti-tumor response with a localized release of immune checkpoint blocking antibody (anti-PD-L1 blocking antibody (aPDL-1) in *in vitro* and immunogenic *in vivo* mouse models. Tumor recurrence prevention after primary resection is the therapeutic advantage of this chemo-immunotherapy [63].

## 5. Translation to the Clinic

With enormous potential for therapeutic applications, several hydrogel formulations have crossed the barriers of *in vitro*/pre-clinical studies and found their way into the market. Some of them are still in the clinical study phases. Hydrogels have evolved over time to one of the best and the most versatile drug delivery platforms. Table 2 lists the widespread practical applications of the hydrogel concept that have been translated to the clinical level.

## 6. Conclusions

Hydrogels offer a versatile platform for the therapy of several diseases including cancer and diabetes. The water-loving nature of hydrogels and the ability to shrink and swell depending on several environmental cues or the mere presence of water is attractive for drug delivery applications. They have a high degree of porosity and the polymers building them could be cross-linked to varying degrees by adjusting their densities. With a physical structure highly amenable to modification in several ways, the hydrogel applications are not just limited to targeted drug delivery. They also find applications in hygiene products, wound dressings, contact lenses and tissue engineering.

Recent developments of hydrogels in the field of targeted drug delivery have been tremendous. They are modified with targeting ligands and diverse polymer types that confer very interesting properties on them for drug delivery. Ophthalmic drug delivery is an area seeing significant impact in therapy from hydrogels. From comfortable contact lenses to biodegradable drug delivery the applications in eye care have been enormous. They are 90% water, provide steady state drug release over days or months, deliver small molecules or large proteins, are fully absorbed in delivery and remain visible during monitoring [51]. 

Noteworthy is the application of pH responsive hydrogels for cancer therapy and glucose responsive hydrogels for diabetes. The use of modified stem cell membranes for targeted delivery is a very recent and attractive strategy for drug delivery. These membranes coated on hydrogels (nanogels) loaded with drugs are highly specific to the disease site in cancer and are highly bio-compatible. 

Immunotherapy platforms using hydrogels are very significant in cancer therapy. Hydrogels enabling localized delivery of antibodies and other immune-regulatory molecules at cancer sites are promising drug delivery vehicles for cancer therapy. Gastro-retentive drug dosage forms (GRDDFs) are versatile drug delivery platforms for intestine and they offer the advantages of adjusting the nanoparticle size to facilitate retention of the active ingredient in the GI tract for as long as required.

Though the hydrogel-based drug delivery was originally influenced by the hydrophobicity of the drugs, several improvements have been made recently including development of cyclodextrins modified to accommodate the hydrophobic drug sufficiently. Adhesive and conductive patches developed using hydrogels are useful in cardiac repair and vascularization. Remotely controlled motility of hydrogel (mimicking motion of a magbot) and the QD DNA hydrogels are novel ideas to facilitate targeted drug delivery.

As discussed in the paper, though there are several hydrogel formulations in clinical use, there is always scope for improvement and modification of hydrogels to enhance their applications. With subtle modifications to the existing ones, the hydrogels could become superlative drug delivery vehicles surpassing the disadvantages and current limitations with the use of several conventional delivery forms and provide promising results for therapy of several illnesses.

## Figures and Tables

**Table 1:** Different types of hydrogel products administered via different routes of administration [38,39,40,41,42,43,44,45,46,47,48,49,50].

Routes of Administration	Shape	Typical Dimension
Peroral	Spherical beads	1 μm to 1mm
	Discs	Diameter of 0.8 cm and thickness of 1 mm
	Nanoparticles	10–1000 nm
Rectal	Suppositories	Conventional adult suppositories dimensions (length ≈ 32 mm) with a central cavity of 7 mm and wall thickness of 1.5 mm
Vaginal	Vaginal tablets	Height of 2.3 cm, which of 1.3 cm and thickness of 0.9 cm
	Torpedo-shaped pessaries	Length of 30 mm and thickness of 10 mm
Ocular	Contact lenses	Conventional dimensions (typical diameter ≈ 12 mm)
	Drops	Hydrogel particles present in the eye drops must be smaller than 10 μm
	Suspensions Ointments	N/A
	Circular inserts	Diameter of 2 mm and total weight of 1 mg (round shaped)
Transdermal	Dressings	Variable
Implants	Discs	Diameter of 14 mm and thickness of 0.8 mm
	Cylinders	Diameter of 3 mm and length of 3.5 cm

Reprinted (adapted) with permission from [3]; the article is open access and the content reusable. The article is licensed by Creative Commons and the link to the license is available at https://creativecommons.org/licenses/by/3.0/.

**Table 2:** Examples of hydrogels translated to clinical use [64,65,66,67,68].

Product	Type of Hydrogel	Therapeutic Application	Drug Delivered
**Sericin**	**Dextran**	**Optically trackable drug delivery system for malignant melanoma**	**Doxorubicin**
**Hyalofemme/Hyalo Gyn**	**Carbomer propylene glycol, Hyaluronic acid derivative**	**Vaginal dryness, estrogen alternative**	**Hyaluronic acid derivative**
**Dextenza**	**Polyethylene glycol**	**Intra-canalicular delivery for post-operative ophthalmic care**	**Dexamethasone**
**Regranex**	**Carboxymethyl cellulose**	**Diabetic foot ulcer**	**Recombinant human platelet derived growth factor**
**muGard**	**Mucoadhesive**	**Oral lichen planus**	
	**2% Poloxamer**	**Cervical cancer recurrence**	**Carboplatin**
